# Complete High Thoracic Spinal Cord Injury Causes Bowel Dysfunction in Mice

**DOI:** 10.1089/neu.2024.0277

**Published:** 2025-04-03

**Authors:** Olivia H. Wireman, Ellie L. Sams, Lynnet E. Richey, Gabrielle V. Hammers, Andrew N. Stewart, William M. Bailey, Samir P. Patel, John C. Gensel

**Affiliations:** ^1^ Department of Physiology, Spinal Cord and Brain Injury Research Center, College of Medicine, University of Kentucky, Lexington, Kentucky, USA.; ^2^ Department of Neuroscience, Spinal Cord and Brain Injury Research Center, College of Medicine, University of Kentucky, Lexington, Kentucky, USA.

**Keywords:** bowel, constipation, dysfunction, SCI, transection

## Abstract

Bowel dysfunction, is a prevalent and life-impacting comorbidity of spinal cord injury (SCI) with no long-term treatment available. SCI-induced colon changes including motility and fibrosis are understudied as are strategies to address SCI bowel dysfunction. This need remains partly due to the lack of a mouse model that recapitulates the human condition. We hypothesized that a high thoracic spinal transection in mice would trigger bowel dysfunction with coincident colon pathology similar to humans and rats after SCI. We observed bowel dysfunction as increased fecal pellet numbers within the colon, smaller pellet size, and decreased motility. Fecal pellets numbers in the colon increased significantly in SCI animals versus sham (laminectomy only) injuries by 4 days postinjury (dpi) and persisted to 7 and 21 dpi. The number of pellets expelled (fecal output) significantly decreased in SCI versus sham animals at both 7 and 20 dpi. Pellet size was significantly decreased in SCI animals at 7 and 14 dpi, collectively indicative of decreased motility with SCI. SCI caused non-significant reductions in colonic motility (bead expulsion assay) at all three timepoints. Through *ex vivo* myograph analyses of live colon sections, we detected significant increase in the maximal contractility of the circular musculature from both the proximal and distal colon after SCI at 21 dpi. At the same time point, distal colons displayed significant collagen deposition in the musculature after SCI. Collectively, these findings demonstrate bowel dysfunction immediately after injury that continues in the distal colon over time. Establishing this mouse model enables further interrogation using transgenic models.

## Introduction

Bowel dysfunction is a common and life-impacting comorbidity associated with spinal cord injury (SCI).^1–3^ Bowel dysfunction after SCI, otherwise termed neurogenic bowel, can present as persistent constipation or incontinence depending on the severity and location of SCI.^4^ There is currently limited treatment for bowel dysfunction after SCI beyond addressing symptoms of constipation which involves taking oral laxatives, the use of suppositories, and digital rectal stimulation.^1,5^ Surveys of those living with SCI have repeatedly shown that regaining control of bowel function is a top priority.^6–10^

The mechanism behind bowel dysfunction after SCI is not fully understood. Central control to sympathetic and lower parasympathetic pathways, which innervate the intestines, are disrupted in injuries above the T5 spinal level. Loss of sympathetic outflow disrupts circular muscle contraction in cats.^11^ Indeed, clinically, SCI induces aberrant contractile patterns in the colon including decreased compliance and failure to initiate peristaltic contractions in response to a meal.^12–14^ The prevalence of difficulty with defecation, gastrointestinal pain, hemorrhoids, incidences of autonomic dysreflexia, and other complications associated with neurogenic bowel increases over time after SCI.^15^ Clinical studies and studies in pre-clinical models of SCI report pathological changes within the colon itself. Both humans and rats experience a similar drop in colonic contractility after injury and increased collagen deposition in the colonic musculature.^16–19^

The historical use of rats, instead of mice, to examine colon dysfunction after SCI places limitations on mechanistic investigations due to the comparatively limited availability of transgenic models and molecular tools. Studies of bowel dysfunction after T9 contusion in mice report acute changes in the microbiome and increased gastrointestinal permeability.^20^ However, there remains a significant need to interrogate changes in the colon of mice after high-level thoracic injury. Cervical and high thoracic injuries are known to induce the highest incidence of constipation after SCI in humans.^4^

Here, we sought to validate a mouse model of bowel dysfunction after high thoracic level 3 (T3) spinal transection (T3 Tx) using measurements of function and pathology. We hypothesized that a T3 Tx in mice would induce constipation alongside collagen deposition in the colon wall. We measured constipation using fecal output measures (pellet count and weight) alongside pellet count in the colon. Motility and contractility were measured in the colon using the bead expulsion assay and myography, respectively. Collagen deposition within the colon wall was interrogated histologically using Masson’s tri-chrome structural staining. Our findings indicate that mice develop constipation after T3 Tx as indicated by decreased fecal output and pellet size and increased pellet retention. Findings from the bead expulsion assay and myograph suggest this constipation could be owed in part to decreased motility in the distal colon and increased tone at the most distal portion of the colon. Collagen deposition was most notable in the distal portion of the colon. By characterizing this model of bowel dysfunction in mice we increase the ability to investigate the mechanisms behind SCI-induced progressive bowel dysfunction. The goal of this work is to facilitate the identification of targets for interventions that could improve the long-term functionality of the colon over the lifetime of a person with SCI.

## Materials and Methods

### Animal care and housing conditions

All procedures were approved by the University of Kentucky Institutional Animal Care and Use Committee. Female C57BL/6J (The Jackson Laboratory) mice between the ages of 4 and 6 months were individually housed throughout the experiment. Due to the severity of the T3 Tx injury, female mice were used to avoid attrition common when using male mice, especially with manual bladder expression.^21^

### Injury model

Mice were injured with either a T3 complete transection or sham (laminectomy) surgery. Animals were anesthetized using isoflurane, first at a flow rate of 500 mL/min at 3.0% isoflurane and then adjusted down and maintained at 200 mL/min at 2.0% isoflurane using a Somonoflo, a low flow electronic vaporizer (Kent Scientific; SF-01). To start, the area below the ears to the bottom of the scapula was shaved to remove hair from the surgical site. This area was then sterilized first with isopropyl alcohol followed by betadine. A midline skin incision was made starting just above the scapula and extending 2–3 cm. The exposed fat pad was then blunt dissected using pointed scissors until the underlying musculature was exposed. The musculature was then carefully pulled to the side using forceps, diligently avoiding arteries. Once the laminae were cleared of musculature, the T2 prominent spinal process was firmly gripped with forceps.^22^ Both sides of the T3 lamina were dissected using iridectomy scissors and the spinous process was removed to expose the T3/T4 spinal levels. The cord was then bathed in lidocaine for 1 min, which was then suctioned off prior to transection. A scalpel (size 11) was then used to transect the cord in a sweeping motion away from the surgeon. This step was repeated until there was a clear space visible between the two stumps of the cord. A small piece of dissolvable gel foam (Ethicon; 1975) was placed in the spinal canal between the two stumps to assist coagulation. The muscular layer was then sutured together using size 5-0 absorbable sutures (Esutures.com; um213) and the skin was sutured using size 2-0 nonabsorbable sutures (VWR; 95060-062). The spinal cord of the sham animals was exposed and bathed in lidocaine and then closed in the same way with no transection. Both sham and injured animals were immediately treated with slow-release buprenorphine (0.05–0.1 mg/kg subcutaneously) and antibiotics (enrofloxacin; 2.5 mg/kg/day). Both groups were then treated with antibiotics daily over the course of the first 5 days after injury. Injured animals were maintained on a water heating pad (38°C) and cages with minimal bedding and a white alpha pad for the duration of the study. Injured animals’ bladders were expressed twice daily for the duration of the experiment.

### Caloric/water intake and body weight

Food and water intake was measured daily alongside body weight. Body weight was recorded after expressing the bladder and before the injection of antibiotics. Water intake was measured by decanting the water bottle into a 1 L graduated cylinder and calculating the difference from the previous day. Caloric intake was calculated by converting the weight consumed of standard laboratory chow (3.1 kcal/g), fruit-flavored cereal (i.e., fruit loops) (3.8 kcal/g), and diet gel (1.06 kcal/g; ClearH_2_O; 76A) into kilocalories using conversions given by the suppliers. Water loss by evaporation from the diet gel over 24 h made it impractical to weigh again. Instead, the percentage consumed was estimated and used to calculate the weight consumed.

### Fecal output and water content

Fecal output was modified from previous protocols.^23,24^ Briefly, tests were all started between 9 and 10 AM to control for changes in digestion over the course of the day. Mice were placed in individual cages with no bedding and a flat, white cotton pad (Alpha Pad; LBS Serving Biotechnology) for easy visualization of fecal pellets. Pellets were collected at 5-min intervals for 1 h. and placed immediately in an airtight Eppendorf tube. The pellets were counted and weighed to obtain fecal pellet weight.

### Fecal pellet count in the colon

Animals were overdosed using ketamine (150 mg/kg) and xylazine (15 mg/kg) and transcardially perfused using ice-cold phosphate-buffered saline (PBS). The colon, starting at the cecum, was gently pulled from the mesentery and excised at the anus. The number of fecal pellets was then recorded from the most distal point of the cecum to the end of the dissected colon. Pellets that fell out during dissection were also counted.

### Bead expulsion assay

This protocol was adapted from a previously published protocol.^25^ Animals were lightly anesthetized with isoflurane (Covetrus; 029405; 200 mL/min at 2.0–3.0%). A soft glass rod with rounded, fire-polished end (3 mm diameter) was dipped in lubricant and used to lubricate the opening to the anus. A 3 mm glass bead was then inserted 2 cm into the colon via the anus using the glass rod. The animal was then removed from isoflurane and placed in a clean cage with a wire bottom. The animals were then checked at least every 5 min for expulsion of the bead. After 200 min, if the bead had not been expelled, the abdomen was massaged in a downward motion, and extra lubricant was used to facilitate the passing of the bead. Animals in which the bead could not be manually expelled were left for up to 8 h. to pass the bead. Animals which did not pass the bead within 8 h were humanely euthanized. Two animals were euthanized at 20 dpi because of bowel perforation by the glass rod/bead. The data from these animals at the two other time points were included as the animals were otherwise healthy leading up to the 20 dpi time point.

### Collagen staining and quantification

Colons were removed whole from the distal point of the cecum to the anus and placed immediately into 4% paraformaldehyde for approximately 2 h. Segments of the colon were embedded in paraffin from both the proximal and distal ends. Tissue was sectioned at a 6 µm thickness using a microtome and hot water bath. They were mounted onto positively charged slides (VWR; 48311-703). Masson’s tri-chrome was used for collagen visualization in the context of musculature and nuclei. Tissue was first cleared using xylene and rehydrated with a gradient of ethanol. Antigen retrieval was then done using Bouin’s fluid (VWR; RC112032) at 56°C for 15 min then left at room temperature overnight. The next day, in this order, the slides were rinsed in running tap water for 5–10 min, submerged in a working solution of Weigert’s Iron Hematoxylin (Millipore Sigma; HT1079-1SET) for 5 min, washed in running tap water for 5 min, rinsed in deionized water with 10 dips, submerged in Biebrich Scarlet-Acid Fuchsin (0.9% Biebrich Scarlet VWR 97061-228, 0.1% acid fuchsin VWR 200005-658, 1.0% acetic acid VWR 97064–482) for 5 min, rinsed in deionized water with 10 dips, submerged in phosphotungstic-phosphomolybdic acid (Electron Microscopy Sciences; 26367-05) for 5 min, submerged in a working solution of Aniline Blue (2.4% Aniline Blue Electron Microscopy Sciences 26381-06, 2% acetic acid), submerged in 1% acetic acid for 2 min, rinsed in deionized water with 10 dips, and dehydrated through a gradient of ethanol and xylene.

All slides were coverslipped with Permount (Fisher Scientific; SP15-500) and imaged using a slide scanner (Axioscanner Model Z1, Carl Zeiss AG; Oberkochen, GE). Collagen density was quantified in the circular muscular layer specifically, which was defined as the area outside the lamina propria and inside the collagen line separating longitudinal and circular muscle, surrounding the myenteric nervous system. Tracings around the entire circular muscular section were done in two sections taken within 100 µm of each other. The area of each stain was determined using Halo software (v3.6.40134.137 Indica Labs; Albuquerque, NM, USA), and the density was determined by dividing the area of Aniline Blue positive area by the Biebrich Scarlet positive area.

### Myography

Animals were euthanized using 100% CO_2_ at a flow rate of 30–70% for at least 1 min past the secession of respiration followed by cervical dislocation. Mice were then transcardially perfused with ice-cold PBS. The colon was dissected from just below the cecum to the anus and flushed with ice-cold PBS to remove fecal waste. Physiological saline solution (PSS) and high potassium PSS (KPSS) were prepared ahead of time according to the manufacturer’s instructions (Automated Multi Myograph System—630MA Danish Myo Technology). The colon tissue was placed whole into room temperature PSS. Immediately prior to placing the tissue on the myograph, a 2 mm wide ring was carefully cut from both the proximal and distal end of the colon using a repurposed mouse spinal cord matrix (Fisher Scientific; 50-192-8517). Each section was mounted onto the myograph immediately after excision to prevent curling of the tissue. Following the manufacturer’s instructions (Automated Multi Myograph System-630MA Danish Myo Technology), the passive tension was set for each tissue segment by distending in a stepwise fashion until a standard passive force of 12.3 kPa was reached. The maximal contraction of the tissue was elicited using KPSS twice with two washes of PSS between. Data were recorded and exported using LabChart v8.1.27.

### Experimental design

Table 1 documents the animals used in each experiment along with appropriate animal and data exclusions. Note that initial experiments examining pellets in the colon and collagen deposition were done at 4, 7, and 21 dpi. It became clear that the residual effects of spinal shock were present at 4 dpi with severe constipation evident by pellet count in the colon. Fecal output measurements were attempted at 4 dpi; however, most injured animals would not expel pellets within the hour of observation. Moving forward with refined measures of bowel function, including bead expulsion and myography, the 7 dpi was selected as the earliest timepoint to avoid the severe disruption associated with the initial spinal shock. For experiments using two independent variables, including weekly changes in food and water intake, a two-way Analysis of Variance (ANOVA) was employed. A main effect was found allowing for the use of Fisher’s least significant difference (LSD) test to analyze the differences between groups at individual time points. As appropriate, a one-way ANOVA was performed for data comparisons among three or more groups. The Brown–Forsythe test to account for variability in standard deviation across groups and the Kruskal–Wallis test in cases of nonparametric data were used as needed. Dunnett’s post hoc analyses were performed to analyze differences across groups compared to sham. For variables measured over time, a mixed-effects model, which allows for missing data points, was first used to determine the main effect between groups and interactions. If a main effect was found, a Fisher’s LSD test was used for post hoc analysis. Daily measurements taken for body weight were analyzed using simple linear regression to understand differences between rates of change. For all outcomes, sham groups were divided among time points to ensure there was no effect of analgesics or antibiotics. No differences were found between sham groups for measures taken only once, thus shams were combined into one group for these outcomes. Shams remain split for each timepoint for repeated measures. All statistical analysis was run using GraphPad Prism Version 10.2.3. with advice from the statistician (Dr. Greg Hawke) at the University of Kentucky Department of Statistics.

**Table 1.** Experimental Design and Exclusions

Assay(s)	Sample sizes original (final)	Data included/excluded
Food/water intake and weight (daily/ 3 weeks)	Sham *n* = 5 Injured *n* = 8 (7)	One mouse from the SCI group did not survive to end-point, and all data were excluded.
Fecal output (7, 14, and 20 dpi)	Sham *n* = 23 7 dpi *n* = 14 14 dpi *n* = 11 21 dpi *n* = 11 (10)	One mouse from the SCI group was humanely euthanized prior to the end-point. All data were excluded from that animal. Data for three injured animals were accidentally not recorded on 7 and 14 dpi.
Pellets in the colon (4, 7, and 21 dpi)	Sham = 26 4 dpi *n* = 7 (6) 7 dpi *n* = 7 (4) 21 dpi *n* = 22 (20)	One, three, and two injured animals did not survive to end-point in the 4, 7, and 21 dpi groups, respectively. All data from animals that did not make it to the end-point were excluded.
Bead expulsion (7, 14, and 20 dpi)	Sham *n* = 12 7 dpi *n* = 8 14 dpi *n* = 8 20 dpi *n* = 8 (6)	Two injured animals did not survive the final bead expulsion testing. The animals were healthy leading up to the last day of bead expulsion testing.
Myograph (7 and 21 dpi)	Sham *n* = 10 7 dpi *n* = 7 (4) 21 dpi *n* = 7 (6)	Three injured animals in the 7 dpi group and one in the 21 dpi group did not survive to the end-point. All data from these animals were excluded.
Collagen deposition (4 and 21 dpi)	Sham *n* = 8 4 dpi *n* = 6 (5) 21 dpi *n* = 9 (8)	One injured animal from both the 4 and 21 dpi groups did not survive to end-point. All data from these animals were excluded.

SCI, spinal cord injury.

## Results

### T3 transection decreases food intake and drives persistent weight loss

**FIG. 1.**
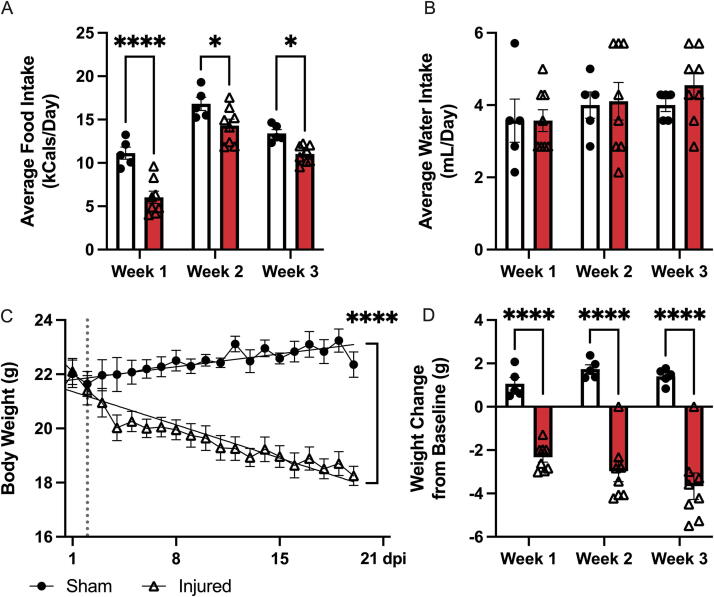
Changes in body weight and food and water intake after T3 transection. The daily food and water intake was recorded for each animal (A and B). **(A)** Injured animals consumed significantly less daily kCal than sham-injured animals throughout the study (*p* < 0.0001 week 1; *p* < 0.05 weeks 2 and 3). **(B)** There was no significant difference in daily water consumption between groups. Body weight was measured before surgery and daily thereafter (C and D). **(C)** The surgery is indicated by the vertical dotted line. Injured animals showed a dramatic decrease in weight over time while sham animals steadily maintained weight. The rate of change for each group was depicted using a simple linear regression. The slope of the two lines was significantly (*p* < 0.00001) different. **(D)** The weight change from baseline was calculated for each animal weekly and averaged across groups. Animals with T3 SCI lost increasingly more weight over time relative to sham-injured controls. A two-way ANOVA with Fisher’s LSD post hoc test was used for all bar graphs. The difference in the rate of change in body weight was analyzed using a simple linear regression. Sham *n* = 5, Injured *n* = 8. ***p < 0.001, ****p < 0.0001. LSD, least significant difference; SCI, spinal cord injury; T3, thoracic level 3.

Following a T3 Tx, mice were monitored daily for food and water intake and body weight. Injured animals ate significantly less kCal each day throughout the study compared to sham-injured animals (main effect of time *p* < 0.0001 and injury *p* < 0.0001; Week 1 *p* < 0.0001 and Week 2 and 3 *p* < 0.05) (Fig. 1A). Water intake (mL) remained consistent between the injured and sham groups throughout the entire study (Fig. 1B). Body weight of the T3 Tx group dropped significantly from baseline over the course of 3 weeks compared to sham controls (*p* < 0.0001) (Fig. 1C). The change from baseline at the end of each week was calculated. T3 Tx animals lost significantly more weight compared to sham animals at all timepoints postinjury (main effect of injury *p* < 0.0001; Weeks 1, 2, and 3 *p* < 0.0001) (Fig. 1D).

### T3 transection induces bowel dysfunction

**FIG. 2.**
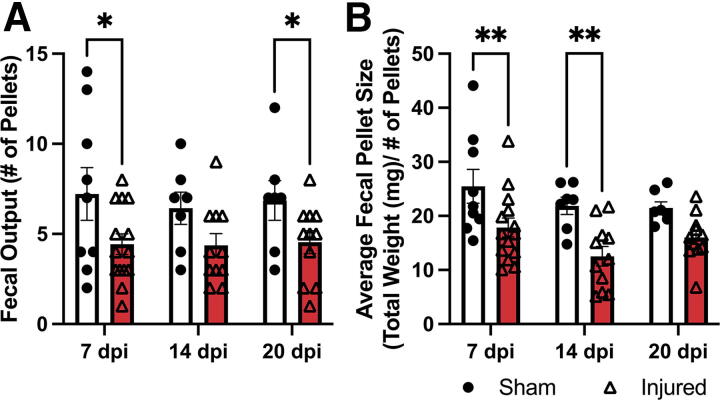
T3 transection triggers long-term bowel dysfunction. **(A)** Animals receiving SCI expelled significantly fewer pellets at 7 and 20 dpi (*p* < 0.05) compared to sham-injured animals. This trend persisted at 14 dpi (*p* = 0.065). **(B)** The average pellet size expelled decreased significantly in injured versus sham animals at 7 and 14 dpi (*p* < 0.01). Only animals that survived to the end-point were included in the data. Data were analyzed using a mixed-effects model with Fisher’s LSD for post hoc comparisons. Sham *n* = 23, 7 dpi *n* = 14, 14 dpi *n* = 11, 21 dpi *n* = 11. *p < 0.05, **p < 0.01. LSD, least significant difference; SCI, spinal cord injury; T3, thoracic level 3.

Fecal pellets collected from animals over a 1 h observation period were used to determine total fecal output and weight. Significantly fewer pellets were expelled by SCI versus sham animals (main effect of injury *p* < 0.01). These effects were significant at 7 and 20 dpi (*p* < 0.05, both) with the decreased trend consistent at 14 dpi (*p* = 0.065; Fig. 2A). On average, fecal pellets were also significantly smaller in injured versus sham animals (main effect of injury *p* < 0.01) with significant group differences at 7 and 14 dpi (*p* < 0.01, both; Fig. 2B). There was a nonsignificant decrease in fecal water content in injured versus sham animals at 21 dpi (unpaired *t*-test *p* = 0.0965; data not shown).

**FIG. 3.**
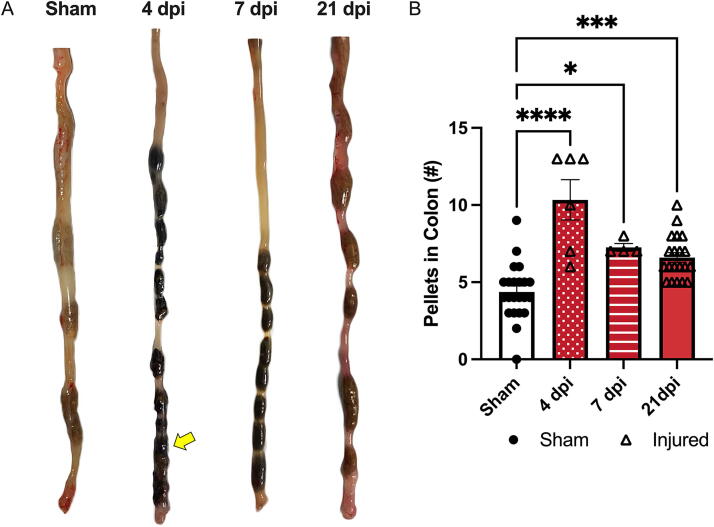
T3 transection increases pellet retention in the colon. **(A)** Images show dissected colons with the most proximal point on the top and distal on the bottom (images are approximately scaled). There was evidence of impaction at 4 dpi (indicated by the yellow arrow) and increased pellet numbers at 7 and 21 dpi. **(B)** Quantification of these images showed a significant increase in the number of pellets in the colon at 4, 7, and 21 dpi compared to sham. Animals that did not survive to the end-point were not included. **(B)** was analyzed using a one-way ANOVA with Dunnett’s multiple comparisons test. Sham *n* = 22, 4 dpi, *n* = 6, 7 dpi, *n* = 4, and 21 dpi, *n* = 20. *p < 0.05, ***p < 0.001, ****p < 0.0001.

Significantly more pellets were retained within the colons of SCI compared to sham-injured animals (main effect of injury *p* < 0.0001). SCI animals had significantly more pellets at 4, 7, and 21 dpi compared to sham (*p* < 0.0001, *p* < 0.05, and *p* < 0.001, respectively; Fig. 3B). The image in Figure 3A shows an increased number of pellets in the large colon postmortem and evidence of fecal impaction in the most distal portion at 4 dpi (indicated by the yellow arrow). Taken together, these findings indicate that mice subjected to a T3 Tx experience constipation-like bowel dysfunction.

### Decreased colonic motility and increased rectal tone

**FIG. 4.**
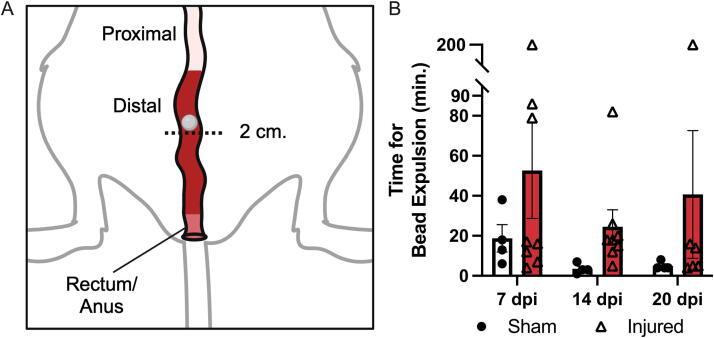
Decreased distal colon motility in injured versus sham animals. Bead expulsion was used as a measure of distal colonic motility by recording the time taken for a glass bead inserted 2 cm. into the colon to exit the anus. **(A)** A diagram shows the location of the glass bead which was inserted 2 cm. past the anus into the distal colon. **(B)** The injured group, on average, took more time to pass the bead at all timepoints (main effect of injury *p* = 0.0988). The bead was manually expelled after 200 min. Data were analyzed using a mixed-effects model. Sham *n* = 12, 7, and 14 dpi, *n* = 8, 21 dpi, *n* = 6.

Motility in the distal colon was specifically measured using colon bead expulsion. Figure 4A depicts the relative location of the bead within the distal colon. Dysfunction within the distal colon and rectum are thus being measured with this assay. The time required to expel the glass bead increased, nonsignificantly, in injured animals compared to sham (main effect of injury *p* = 0.0988) (Fig. 4B). Increased transit time of the glass bead indicates decreased motility in the distal colon and rectum of T3 Tx mice compared to sham.

**FIG. 5.**
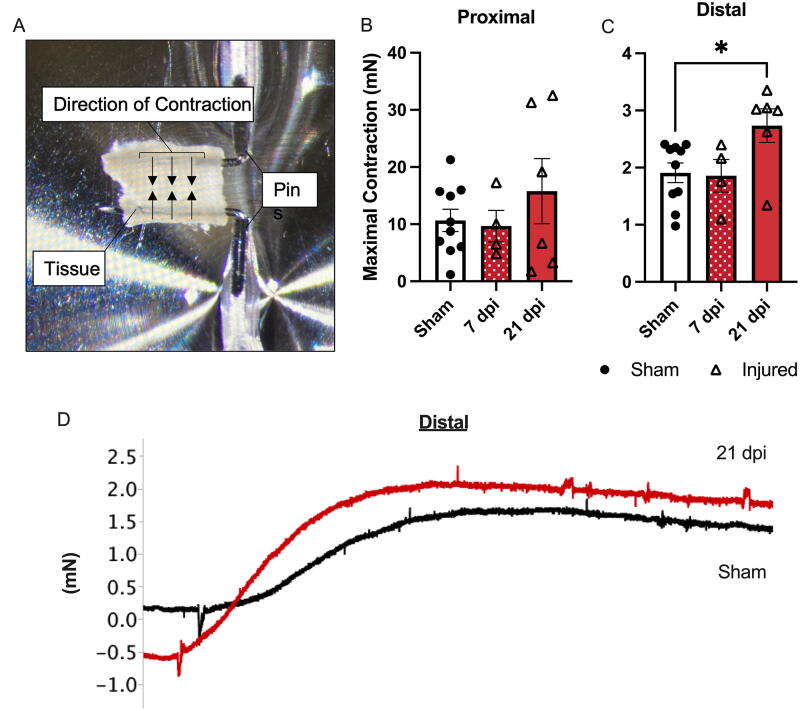
Increased maximal contraction in the distal colon of T3 Tx animals. **(A)** An image of the myograph setup shows a 2 mm wide piece of colon tissue mounted on two myograph pins. Arrows indicate the direction of force when the tissue is stimulated with a potassium-high solution (KPSS). **(B)** A significant increase in the maximal contraction within the distal colon was found at 21 dpi (*p* < 0.05) with a similar trend in the proximal colon. **(C)** Representative traces were taken from the distal colon myograph with sham in black and an animal 21 dpi in red. **(D)** Representative traces were taken from both a sham (black) and an injured animal (21 dpi, red) immediately following the addition of the high potassium solution. A steeper, higher increase in force (mN) is evident in the injured compared to sham animals. **(B)** was analyzed using Brown–Forsythe and Welch ANOVA with Dunnett’s multiple comparisons test. C was analyzed using a one-way ANOVA with Dunnett’s multiple comparisons test. Sham *n* = 10, 7 dpi, *n* = 4, 21 dpi, *n* = 6. *p < 0.05. T3 Tx, thoracic level 3 spinal transection.

To gain even more resolution on the nature and location of this dysfunction, the maximal contraction of proximal and distal colonic circular muscle segments was determined using myography. The image in Figure 5A shows the myograph setup, with a 2 mm. segment of colon mounted on pins connected to force transducers. The direction of contraction, facing inward, is indicated by the arrows. The tissue was continuously bathed in a solution of PSS and aerated (not able to be shown). The maximal contraction was calculated by subtracting the minimum force measurement from the maximum measurement after the application of KPSS. The maximal contraction was significantly higher (*p* < 0.05) at 21 dpi in T3 Tx animals in the distal colon compared to sham (main effect *p* < 0.05) (Fig. 5C). No other timepoints in the proximal or distal colon showed a change in maximal contractility compared to sham (Fig. 5B and C). Representative traces (Fig. 5D) of maximal contractions taken from either sham or T3 Tx 21 dpi animals show an upward slope following the application of KPSS with the change in force greater for the T3 Tx animal at 21 dpi (red) compared to sham (black).

### T3 transection increases collagen in the distal colon

**FIG. 6.**
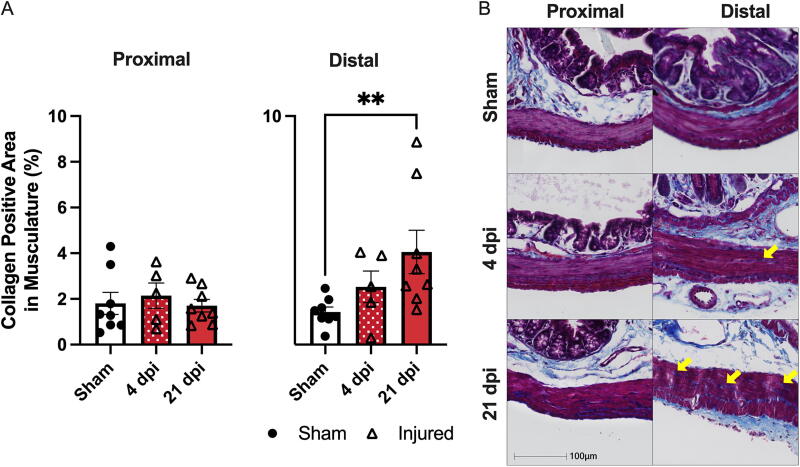
T3 transection triggers collagen deposition in the distal colon at 21 dpi. **(A)** No change in collagen density was seen in the proximal colon (Main effect *p* = 0.791). Collagen density in the circular muscle of the distal colon increased significantly by 21 dpi (*p* < 0.01). **(B)** Representative images of the colon musculature stained with Masson’s tri-chrome show the proximal and distal colon in sham, 4 dpi, and 21 dpi animals (pink—musculature, blue—collagen, and purple—nuclei). Yellow arrows indicate bands of collagen deposited in the circular muscle. A was analyzed using one-way ANOVA with Dunnett’s multiple comparison’s test. B was analyzed using the Kruskal–Wallis test with Dunn’s multiple comparisons. Sham *n* = 8, 4 dpi, *n* = 5, 21 dpi, *n* = 8. **p < 0.01.

Using Masson’s tri-chrome, collagen density in the circular muscle layer was visualized and quantified using Halo Analysis software. The density of collagen deposition in the distal colon significantly increased in T3 Tx animals compared to sham at 21 dpi (*p* < 0.005; main effect *p* < 0.01) (Fig. 6B). There was no change in collagen density in the proximal colon at either 4 or 21 dpi (Fig. 6A). The image in Figure 6C shows collagen (blue), musculature (pink), and nuclei (purple). The yellow arrows indicate the blue bands of collagen within the circular musculature. The number of these bands increases significantly by 21 dpi in the distal colon.

## Discussion

We observed that T3 spinal cord transection in mice induces constipation-like bowel dysfunction alongside collagen deposition in the colon wall. To understand this model, in mice, we first recorded daily food and water intake in addition to weight. T3 Tx animals continued to lose weight over time after injury alongside decreased caloric intake. Changes in bowel function, as measured by fecal output and pellet size, were immediate, starting at 7 dpi, and persisted out to 20 dpi. Injured animals, at most time points, were expelling smaller and less fecal pellets than sham controls. Logically, more pellets were found, postmortem, in the colons of animals with the injury compared to sham. The bead expulsion assay then showed that injured animals require more time to pass a bead placed 2 cm. into the colon compared to sham, indicating dysmotility in the distal colon. Myography indicated increased tone in the distal part of the colon at chronic time points as the maximal contractility in the distal colon increased significantly at 21 dpi in injured versus sham animals. Finally, collagen deposition within the circular musculature similarly increased at chronic time points after injury. Thus, the present study supports the conclusion that T3 Tx in mice is a viable model of SCI-induced bowel dysfunction, recapitulating functional and pathological changes after injury consistent with rat models and humans.

Indeed, our observations of weight changes, food intake, and fecal output are consistent with previous reports. The initial drop in body weight we observed after SCI is a common occurrence in both humans and rodents, attributed to significant muscle wasting in the paralyzed limbs.^26–28^ However, we observed that food intake was reduced after T3 Tx in mice, an observation also reported in rats with T3 Tx.^26^ It is possible, therefore, that both muscle wasting and decreases in food intake contribute to weight loss in the current study. All animals showed a preference for sugary treats placed in the cage over standard chow. Injured animals ate relatively little chow compared to sham animals, which contributed to decreased caloric intake. A previous study using a T3 Tx in rats found a decreased fecal output when monitored for 6 h,^29^ which is consistent with our current findings in mice. Interestingly, in the current study, we observed significant differences in fecal output with one hour of observation indicating that shorter testing times may be sufficient in this model.

Fecal impaction has been reported after both T3 and T10 Tx in rats in the distal portion of the bowel at 7 dpi.^29,30^ Both of these studies also used myography to look at the differential response of the colon to acetylcholine (ACh) in animals either with or without SCI. Consistent with these previous reports, we observed impaction in our mouse model at 4 dpi with persistent deficits out to 21 dpi. Interestingly, while evidence of impaction is consistent in these published studies across spinal injury levels, the myographical data are conflicting. Previous studies using ACh to elicit contraction of the colon seem to indicate a level-dependent nature of the response to injury. Frias et al. found that a T3 Tx decreased contractility in both the proximal and distal colon in response to ACh.^29^ In contrast, the study using a T10 Tx found an increased contractile response to ACh in the distal colon.^29,30^ However, maximal contractility was not reported in either study. The present study recorded maximal contractility in the presence of a high potassium solution. In contrast to contraction elicited by ACh, maximal contractility of the colon, as elicited by potassium, was increased in injured animals. These contrasted findings could point to an interesting relationship between changes in maximal contractility and desensitization of the colon to ACh. Additional studies are needed to determine the extent to which species, spinal level of injury, and experimental conditions confound myographical interpretation. It is worth noting that previous data from human colon biopsies has shown that after SCI there is a chronic increase in collagen in the musculature of the colon.^18^ These histological findings were recapitulated in a T3 Tx in rats,^19^ and again in the present mouse study. Collectively, our observations of fecal impaction, physiological changes in the distal colon, and increased collagen deposition indicate protracted, SCI-induced, neurogenic bowel.

To the best of our knowledge, bead expulsion has yet to be reported in a rodent model of SCI. Our findings of increased transit time through the distal colon are consistent with other published outcomes indicative of reduced total transit time after SCI. A T3 Tx in rats causes an increase in total transit time,^29^ similar to the increased time to expel a bead found in the current study. However, dramatic variability in the injured group decreased the power of the assay. Furthermore, with additional stress on the animal through isoflurane administration and the risk of bowel perforation, this assay may not be best suited for SCI studies.

Our observation of a sustained difference in body weight between sham and T3 Tx animals is inconsistent with reports in rats. Notably, animals receiving the T3 Tx consumed significantly fewer calories than the sham group during the first week after injury, consistent with previous studies using T3 Tx.^26^ However, in a study using T3 Tx in rats, the drop in body weight in the injured animals reversed after the first week with animals steadily returning to their preoperative weight.^26^ While this weight loss could pose a problem to sustaining the animals past 3 weeks, previous literature has shown mice with T3 Tx can survive for at least 4 weeks.^31^ Decreased food intake during the first week could be an important caveat when considering pathology in the colon. Previous data in other models have shown that fasting in mice for up to 3 days can result in intestinal weight loss and apoptosis.^32^ Further studies looking at the long-term effects of this fasting period are necessary. One of the most dramatic differences between the injured and uninjured animals in this study was the severe weight loss seen in the SCI group. Changes in caloric intake during the first week after SCI could influence bowel outcome measures and merit further study.

One limitation of the current study is the use of only female animals. The rationale was the technical limitations of aftercare associated with male SCI mice (i.e., prolonged bladder care, increased attrition).^21^ Little has been published concerning the role of sex in bowel dysfunction after SCI and few studies use both sexes or power for a comparison between sexes. Clinically, irritable bowel syndrome (IBS) has been shown to present more frequently in women compared to men.^33^ Furthermore, constipation-type IBS tends to prevail over diarrhea in women, while the reverse is true in men.^34^ It is unclear in clinical data whether this same sexually dimorphic pattern is replicated in bowel dysfunction after SCI. Therefore, pre-clinical studies powering for an interaction between sex and bowel dysfunction after SCI are needed to elucidate this area of study.

In summary, here we characterized the acute and chronic changes to the bowel after a T3 Tx in mice. Using functional measures including fecal output, pellets retained within the colon, and bead expulsion, we detected dysfunction at both acute and chronic time points. Myography provided resolution on changes within the distal colon happening at chronic time points that were mirrored by collagen deposition in the distal colon. Understanding bowel dysfunction in a mouse model of SCI using basic functional assays opens the door to studying bowel outcomes with a wider range of mouse models of SCI. Furthermore, a transection model of bowel dysfunction in mice provides a platform to interrogate changes within the colon itself after SCI. Mice provide accessibility to transgenic models and tools for molecular and histological outcomes. The availability of this model could increase the body of research on bowel dysfunction after SCI and move our understanding of this condition toward a treatment.

### Transparency, rigor, and reproducibility statement

We are committed to upholding the principles of transparency, rigor, and reproducibility in our research. We aim to provide clear and comprehensive descriptions of our methods, data, and analysis to facilitate understanding by the scientific community. All raw data generated in this study will be made available through the Open Data Commons for SCI to promote transparency and facilitate further investigation. Further, we have implemented several measures to ensure the rigor of our study design. A power analysis with preliminary data was run (1 − β = 0.80; α = 0.05). Injured animals that did not survive to the end-point were excluded unless otherwise noted throughout the article. Our study was designed with careful consideration of potential biases and confounding factors, including appropriate sample sizes, randomization, and blinding procedures. Finally, we have taken proactive steps to enhance the reproducibility of our study. We have documented all materials, reagents, and protocols used in our experiments to facilitate replication by other researchers. We welcome inquiries from fellow researchers to facilitate the replication of our findings. By adhering to these principles, we strive to contribute to the advancement of knowledge and the development of effective therapies for individuals living with bowel dysfunction after SCI.

## Data Sharing

All raw data were deposited at the Open Data Commons for Spinal Cord Injury ([ODC-SCI]; RRID: SCR_016673). The ODC-SCI is a secure, cloud-based repository platform for the field of SCI research that is designed to share research data. The DOI associated with this work is as follows: https://doi.ord/10.34945/F57K5H.
